# Prognostic value of stress cardiovascular magnetic resonance in asymptomatic patients with known coronary artery disease

**DOI:** 10.1186/s12968-021-00721-8

**Published:** 2021-03-08

**Authors:** Théo Pezel, Thomas Hovasse, Marine Kinnel, Thierry Unterseeh, Stéphane Champagne, Solenn Toupin, Philippe Garot, Francesca Sanguineti, Jérôme Garot

**Affiliations:** 1 Institut Cardiovasculaire Paris Sud, Cardiovascular Magnetic Resonance Laboratory, Hôpital Privé Jacques CARTIER, Ramsay Santé, 6 Avenue du Noyer Lambert, 91300 Massy, France; 2 Division of Cardiology, Johns Hopkins University, Baltimore, MD 21287-0409 USA; 3 Siemens Healthcare France, 93200 Saint-Denis, France

**Keywords:** Cardiovascular magnetic resonance, Stress testing, Ischemia, Asymptomatic, Coronary artery disease, Secondary prevention

## Abstract

**Background:**

Several studies have established the prognostic value of vasodilator stress cardiovascular magnetic resonance (CMR) in broad population of patients with suspected or known coronary artery disease (CAD), but this specific population of asymptomatic patients with known CAD have never been formally evaluated. To assess the long-term prognostic value of vasodilator stress perfusion CMR in asymptomatic patients with obstructive CAD.

**Methods:**

Between 2009 and 2011, consecutive asymptomatic patients with obstructive CAD referred for vasodilator stress CMR were followed for the occurrence of major adverse cardiovascular events (MACE), defined by cardiovascular mortality or recurrent non-fatal myocardial infarction (MI). Uni- and multivariable Cox regressions were performed to determine the prognostic value of myocardial ischemia and myocardial infarction defined by late gadolinium enhancement (LGE) with ischemic pattern.

**Results:**

Among 1529 asymptomatic patients with obstructive CAD, 1342 (87.8%; 67.7 ± 10.5 years, 82.0% males) completed the follow-up (median 8.3 years), and 195 had MACE (14.5%). Patients without stress-induced myocardial ischemia had a low annualized rate of MACE (2.4%), whereas the annualized rate of MACE was higher for patients with mild, moderate, or severe ischemia (7.3%, 16.8%, and 42.2%, respectively; p_trend_ < 0.001). Using Kaplan–Meier analysis, myocardial ischemia and LGE were associated with MACE (hazard ratio, HR 2.52; 95% CI 1.90–3.34 and HR 2.04; 95% CI 1.38–3.03, respectively; both p < 0.001). In multivariable stepwise Cox regression, myocardial ischemia and LGE were independent predictors of MACE (HR 2.80 95% CI 2.10–3.73, *p* < 0.001 and HR 1.51; 95% CI 1.01–2.27, *p* = 0.045; respectively). The addition of myocardial ischemia and LGE led to improved model discrimination for MACE (change in C statistic from 0.61 to 0.68; NRI = 0.207; IDI = 0.021).

**Conclusions:**

Vasodilator stress CMR-induced myocardial ischemia and LGE are good long-term predictors for the incidence of MACE in asymptomatic patients with obstructive CAD.

## Introduction

Despite the decline in the rate of recurrent cardiovascular events over the past decades, recurrence remains a major cause of mortality and morbidity among patients with known obstructive coronary artery disease (CAD) [1, 2]. Risk stratifying patients for recurrent cardiovascular events could be helpful to manage therapeutic strategy for secondary prevention in this population. Whereas the interest of coronary revascularization has been very recently debated in patients with stable CAD [3], secondary prevention therapy is therefore key to decrease the rate of recurrent cardiovascular events in this population. The intensification of secondary prevention therapy is possible, justifying the careful selection of patients with a high residual risk and low therapeutic risk.

In patients with known CAD, the prevalence of myocardial ischemia is estimated around 20% [4]. Furthermore, several studies have shown that asymptomatic patients with myocardial ischemia have at least similar risk for adverse cardiovascular events and mortality as symptomatic patients with typical angina [5, 6]. While risk stratification of asymptomatic patients can be useful in managing secondary prevention, the European and American guidelines do not recommend systematic stress testing in the follow-up of patients with CAD [7, 8], because they are mainly based on studies of symptomatic patients.

Cardiovascular magnetic resonance (CMR) imaging has emerged as an accurate technique to assess ventricular function, the presence of myocardial scar and viability, and myocardial ischemia without ionizing radiation [9–11]. Vasodilator stress CMR has been reported in several studies to be effective to stratify the risk of recurrent cardiac events in secondary prevention [9, 10, 12]. Although several large stress CMR prognostic studies have included asymptomatic patients, targeted prognostic data in those patients with known CAD are very scarce, and dedicated subgroup analysis has not been separately performed [9, 13].

This study aimed to assess the long-term prognostic value of vasodilator stress perfusion CMR in asymptomatic patients with prior obstructive CAD and to evaluate the incremental impact of stress CMR compared to traditional CAD risk factors.

## Methods

### Study population

Between December 2009 and December 2011, we conducted a single-centre longitudinal study with retrospective enrollment of consecutive asymptomatic patients with known obstructive CAD, referred for vasodilator stress perfusion CMR. Known obstructive CAD was defined by a history of percutaneous coronary intervention (PCI), coronary artery bypass graft (CABG) or myocardial infarction (MI), defined by a history of MI on the medical records or presence of significant Q wave on 12-lead electrocardiogram (ECG) in a coronary territory [14]. Exclusion criteria were: (1) any reported cardiovascular-related symptoms such as chest pain or shortness of breath at rest or on exertion 6 months prior to enrollment; (2) contraindication to CMR (e.g., cerebral clips, metallic eye implant); (3) contraindication to dipyridamole; (4) known allergy to gadolinium-based contrast medium; and (5) estimated glomerular filtration rate < 30 ml/min/1.73 m^2^. Clinical data were collected according to medical history and clinical examination on the day of stress CMR. All patients provided informed written consent. The study was approved by the local ethic committee of our institutions and conducted in accordance with the 1964 Declaration of Helsinki. This study followed the Strengthening the Reporting of Observational Studies in Epidemiology (STROBE) reporting guideline for cohort studies.

### Patients follow-up and clinical outcome

The follow-up consisted of a clinical visit as part of usual care (62%) or by direct contact with the patient or the referring cardiologist (38%). A clinical questionnaire with a detailed description of clinical study endpoints was filled out by three senior cardiologists. Data collection was ended on January 2020. The primary endpoint was the occurrence of at least one of the combined major adverse cardiovascular events (MACE) defined as cardiovascular mortality or non-fatal MI. The secondary endpoint was cardiovascular mortality. Clinical event adjudication was based on the follow-up clinical visit or contact, with a consensus reached by two senior cardiologists. Non-fatal MI was defined by typical angina of ≥ 20 min duration, ECG changes, and a rise in troponin or creatine kinase level above the 99 percentile of the upper reference limit [14]. Cardiovascular mortality was defined as sudden cardiac death with documented fatal arrhythmias or any death immediately preceded by acute MI, acute or exacerbation of heart failure, or stroke. All clinical events were defined according to the published standardized definitions [15]. In patients with multiple events, only the first event was considered for event-free survival analysis. Late coronary artery revascularization was defined by a revascularization occurring > 90 days after CMR. For patients who underwent PCI < 90 days after the index examination, peri-procedural events (MI or cardiovascular mortality) [16] were not included in the analysis.

### CMR protocol

The detailed CMR protocol has been published in previous studies [17, 18]. CMR was performed with a 1.5 T CMR system (MAGNETOM Espree, Siemens Healthineers, Erlangen, Germany) with an 18-channel anterior body coil. Long-axis (2-, 3-, and 4-chamber) and short-axis cine images encompassing the left ventricle (LV) from base to apex. Vasodilatation was induced with dipyridamole injected at 0.84 mg/kg over 3 min. At the end of dipyridamole infusion, a 0.1 mmol/kg bolus of gadolinium-based contrast (Dotarem, Guerbet, Paris, France,) was injected at a rate of 5.0 ml/s with an injector (Mallinckrodt Optistar Elite). Stress perfusion imaging was performed using an ECG-triggered saturation-prepared balanced steady state free precession (bSSFP) sequence with the following typical parameters: repetition time/echo time (TR/TE) = 287/1.2 ms, acceleration factor = 2, field of view = 370 × 314 mm, matrix = 224 × 180, reconstructed pixel size = 1.7 × 1.7 × 8 mm. A series of six slices (four short-axis views, a 2-chamber, and a 4-chamber view) were acquired every other heartbeat. Then, theophylline was injected intravenously to null the effect of dipyridamole. Ten minutes after contrast injection, breath-hold contrast-enhanced 3D T1-weighted inversion-recovery gradient-echo sequence was acquired with the same prescriptions to detect late gadolinium enhancement (LGE). Safety was studied with clinical monitoring 1 h after CMR.

### CMR image analysis

The *syngo*.via software (Siemens Healthineers) was used for image display and processing, and Hemolia (Clinigrid Inc., Paris, FR) was used for reporting. LV volumes and function were quantified on the short-axis cine stack. Stress perfusion and LGE images were evaluated according to the 17-segment model of the American Heart Association [19]. The analysis of perfusion images was done visually by two experienced cardiologists (JG and FS) blinded to clinical and follow-up data. Myocardial ischemia was defined as a subendocardial perfusion defect that (1) occurred in at least one myocardial segment, (2) persisted for at least three phases beyond peak contrast enhancement, (3) followed a coronary distribution, and (4) occurred in the absence of LGE in the same segment [13, 20]. The diagnosis of peri-infarction myocardial ischemia surrounding prior MI was confirmed when the perfusion deficit exceeded the limits of LGE by ≥ 1 segment. A transmural perfusion defect in the same area than a subendocardial LGE was not considered as myocardial ischemia. The long-axis perfusion images were used to confirm perfusion defects visualized on the short-axis slices and to assess the apical segment. A myocardial scar was defined by LGE with ischemic patterns defined by subendocardial or transmural LGE [21]. A scar was considered viable if the LGE thickness was < 50% of the total myocardial wall, and nonviable if ≥ 50% [22]. The total number of ischemic segments was calculated for each patient. Mild, moderate, and severe myocardial ischemia were defined as the involvement of 1–2, 3–5, and ≥ 6 myocardial segments, respectively, as already described [10]. LGE was semi-quantitatively assessed using the number of LGE segments.

### Statistical analysis

Continuous variables were expressed as mean ± standard deviation (SD) and categorical variables as frequency with percentage. Follow-up was presented as median and interquartile range (IQR). Differences between patients with and without myocardial ischemia in terms of baseline clinical and CMR characteristics were compared using the Student’s t-test or the Wilcoxon rank-sum test for continuous variables and the chi-square or Fisher’s exact test for categorical variables, as appropriate. Normal distribution was assessed using the Shapiro–Wilk test. Cumulative incidence rates of individual and composite outcomes were estimated using the Kaplan–Meier method and compared with the log-rank test. Data on patients who were lost to follow-up were censored at the time of the last contact. Cox proportional hazards methods were used to identify the predictors of MACE among patients with and without myocardial ischemia. The assumption of proportional hazards ratio (HR) was verified.

The different multivariable models used for adjustment were as follows:

Model 1: used a stepwise forward Cox regression strategy to select the strongest parsimonious set of clinical covariates for MACE and cardiovascular mortality, considering all clinical covariates with a p-value ≤ 0.2 on univariable screening (without the presence of myocardial ischemia and LGE).
Model 2: model 1 + presence of myocardial ischemia and LGE.
Model 3: included the following traditional cardiovascular risk factors: age, male, body mass index (BMI), hypertension, diabetes mellitus, current or previous smoking, dyslipidemia and LV ejection fraction (LVEF).
Model 4: model 3 + presence of myocardial ischemia and LGE.

The discriminative capacity of each model for predicting MACE was determined according to the Harrell’s C-statistic at baseline and after addition of CMR-induced myocardial ischemia and LGE. The additional predictive value of the presence of myocardial ischemia and LGE was calculated by the Harrell’s C-statistic increment, the categorical net reclassification improvement (NRI), and the integrative discrimination index (IDI). NRI and IDI were computed at the end of follow-up using the R package “survIDINRI” [23].

In competitive risk analysis, cumulative incidence functions (CIFs) were used to display the proportion of patients with the event of interest or the competing event (non-fatal MI or cardiovascular mortality) as time progressed. To analyze the effect of baseline predictors on the CIF, we used the Fine and Gray regression model for the subdistribution hazard.

In addition, the prognostic value of stress CMR in different subsamples of clinical interest were investigated by a Forest Plot. A two-tailed p-value < 0.05 was considered statistically significant. Statistical analysis was performed using R software, version 3.3.1 (R Project for Statistical Computing, R Foundation for Statistical Computing, Vienna, Austria).

## Results

### Patients characteristics

**Fig. 1:**
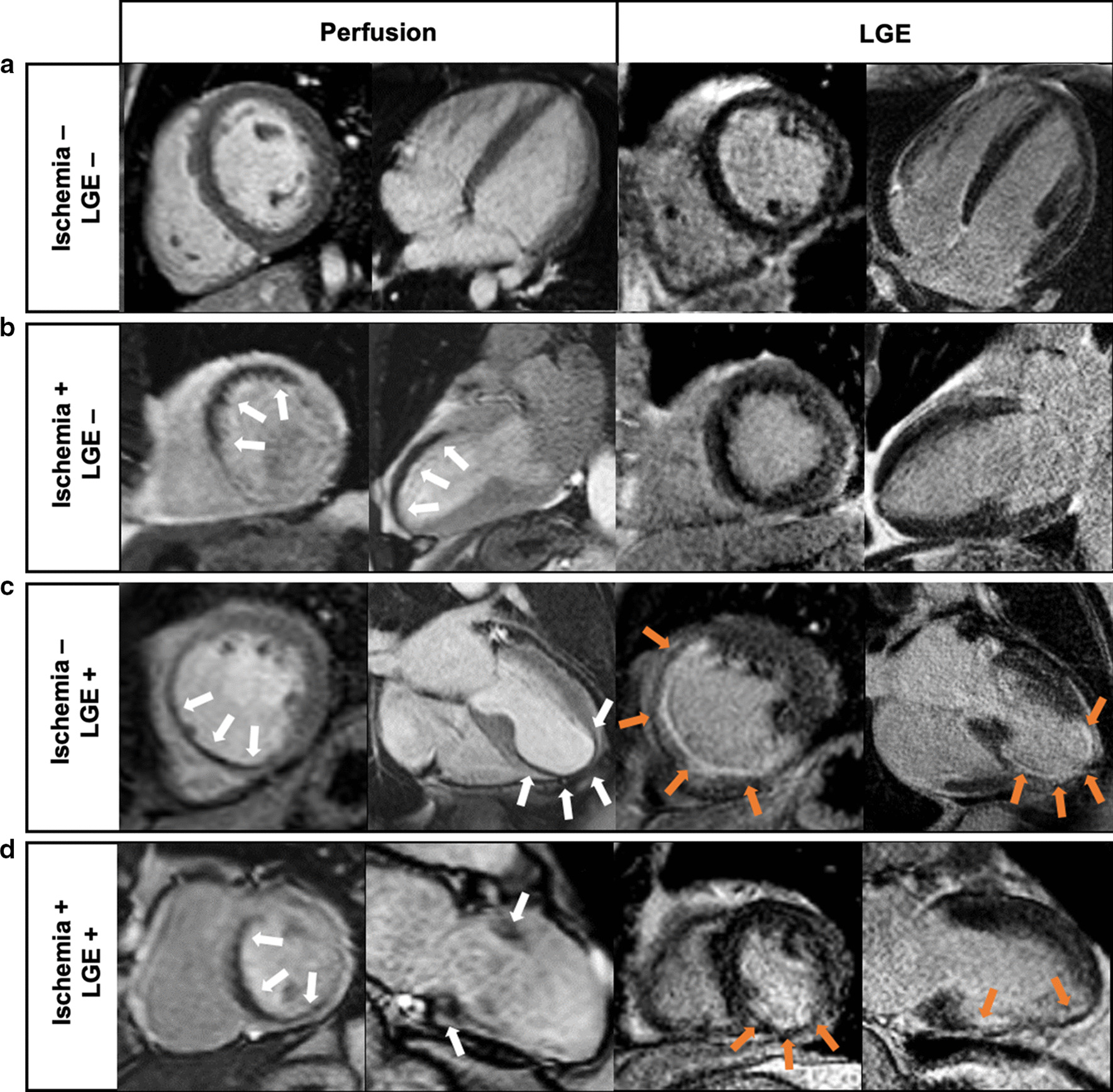
Examples of inducible myocardial ischemia on stress CMR in asymptomatic patients. **a **Normal. 58-year old male with hypertension and history of non-ST elevation myocardial infarctino (NSTEMI) treated by percutaneous coronary intervention (PCI) of the left anterior descending coreonary artery (LAD). Stress CMR revealed no perfusion defect and late gadolinium enhancement (LGE) was negative, ruling out the diagnosis of myocardial ischemia. **b** inducible ischemia. 62-year old female with a history of inferior NSTEMI treated by PCI of the right coronary artery (RCA). First-pass myocardial stress perfusion images revealed a reversible perfusion defect of the anteroseptal wall (*white arrows*) without LGE, indicative of myocardial ischemia suggestive of significant LAD stenosis, confirmed by coronary angiography. **c** myocardial scar without ischemia. 73-year old male with prior anterior STEMI treated by PCI of the LAD. Stress CMR showed a subendocardial antero-septo-apical scar on LGE (*orange arrows*), with a colocalization of the perfusion defect (*white arrows*) and, therefore, no inducible ischemia. Coronary angiography confirmed the absence of significant stenosis. **d** myocardial scar with ischemia. 66-year old male with a history of inferior STEMI treated by PCI of the RCA. Stress CMR showed a subendocardial scar on the inferior wall on LGE sequences (*orange arrows*), and a perfusion defect of the antero-septo-basal wall (*white arrows*) on first-pass perfusion images, indicative of inducible myocardial ischemia. Coronary angiography revealed high-grade stenoses of the LAD

Among the 6,095 individuals referred for dipyridamole vasodilator stress CMR during the inclusion period, 1,529 (25.1%) patients were asymptomatic with prior obstructive CAD. The flowchart of study participants is depicted in Additional file 1. A total of 1342 asymptomatic patients with prior obstructive CAD completed the clinical follow-up and constituted our study cohort. Baseline patient characteristics and baseline CMR data are shown in Table 1. Among these 1342 patients (82.0% males, 67.7 ± 10.5 years), 55.6% had prior MI, 54.8% previous PCI and 32.1% previous CABG. Most subjects were in sinus rhythm (99.6%). The overall study cohort had a mean LVEF of 45.8 ± 12.5%. A MI was diagnosed by LGE with ischemic patterns in 689 (51.3%) patients, and the presence of myocardial ischemia was detected in 376 (28.0%) patients (Fig. 1). Among the 409 diabetics, 151 (36.9%) had myocardial ischemia.

**Table 1:** Baseline and CMR Characteristics of Patients with and without Myocardial Ischemia (N = 1342)

	All patients (N = 1342)	No ischemia (N = 966)	Ischemia (N = 376)	p value
Age (years)	67.7 ± 10.5	67.2 ± 10.7	69.0 ± 9.3	0.003
Males, n (%)	1101 (82.0)	781 (80.8)	320 (85.1)	0.024
Body mass index (kg/m^2^)	27.2 ± 4.0	27.1 ± 3.9	27.5 ± 4.0	0.135
Coronary risk factors, n (%)				
Diabetes mellitus	409 (30.5)	258 (26.7)	151 (40.2)	< 0.001
Hypertension	691 (51.5)	468 (48.4)	223 (59.3)	< 0.001
Dyslipidemia	849 (63.3)	589 (61.0)	260 (69.1)	0.006
Current or previous smoking	324 (24.1)	244 (25.3)	80 (21.3)	0.144
Family history of CAD	431 (32.1)	309 (32.0)	122 (32.4)	0.923
Obesity^a^	310 (23.1)	220 (22.8)	90 (23.9)	0.703
Medical history of CV disease, n (%)				
Prior PCI	735 (54.8)	552 (57.1)	183 (48.7)	0.006
Prior CABG	431 (32.1)	309 (32.0)	122 (32.4)	0.923
Prior MI	746 (55.6)	570 (59.0)	176 (46.8)	< 0.001
Peripheral atheroma	115 (8.6)	64 (6.6)	51 (13.6)	< 0.001
Ischemic stroke	35 (2.6)	28 (2.9)	7 (1.9)	0.379
Pacemaker	2 (0.1)	1 (0.1)	1 (0.3)	0.482
Renal failure^b^	16 (1.2)	12 (1.2)	4 (1.1)	1.000
Heart failure hospitalization	31 (2.3)	24 (2.5)	7 (1.9)	0.631
Indications to stress CMR (multiple possible), n (%)				
PCI or CABG follow-up^c^	1184 (88.2)	830 (85.9)	354 (94.1)	< 0.001
Inconclusive stress test	107 (8.0)	65 (6.7)	42 (11.2)	0.01
Inconclusive CCTA^d^	110 (8.2)	60 (6.2)	50 (13.3)	< 0.001
Cardiac rhythm, n (%)				
Sinus rhythm	1151 (85.8)	818 (84.7)	333 (88.6)	
Sinus rhythm with extrasystoles	185 (13.8)	145 (15.0)	40 (10.6)	0.039
Atrial fibrillation/supraventricular arrhythmias	6 (0.4)	3 (0.3)	3 (0.8)	
LV ejection fraction, %	45.8 ± 12.5	45.1 ± 12.5	47.7 ± 12.5	< 0.001
LV end-diastolic volume index, ml/m^2^	100.2 ± 31.3	102.2 ± 32.1	95.1 ± 28.4	< 0.001
LV end-systolic volume index, ml/m^2^	57.1 ± 27.4	58.9 ± 27.7	52.2 ± 25.8	< 0.001
LV mass, g/m^2^	73.8 ± 7.1	73.6 ± 7.2	73.5 ± 7.1	0.319
RV ejection fraction, %	57.2 ± 11.2	57.1 ± 11.3	57.3 ± 12.9	0.429
Presence of LGE, n (%)	689 (51.3)	502 (52.0)	187 (49.7)	0.018
Presence of viability^e^, n (%)	280 (20.9)	175 (18.1)	105 (27.9)	< 0.001
Number of segments of LGE	2.3 ± 1.9	2.4 ± 1.9	2.2 ± 2.0	0.268
Number of segments of myocardial ischemia	0.7 ± 1.5	0.0 ± 0.0	2.5 ± 1.7	< 0.001
HR at baseline, beats/min	67 ± 12	67 ± 12	71 ± 13	0.512
HR at stress, beats/min	91 ± 9	90 ± 9	96 ± 11	0.069
RPP at baseline (k), mmHg-beats/min	9.1 (7.5–10.6)	9.0 (7.5–10.6)	9.2 (7.5–11.0)	0.621
RPP at stress (k), mmHg-beats/min	10.6 (9.0–12.4)	10.4 (9.0–12.4)	10.9 (9.5–12.8)	0.193

Values are n (%), mean ± SD, or median (interquartile range)

*BMI* body mass index, *CABG* coronary artery bypass grafting, *CAD* coronary artery disease, *CCTA* coronary computed tomography angiography, *CMR* cardiovascular magnetic resonance, *CV* cardiovascular disease, *GFR* glomerular filtration rate, *HF* heart failure, *HR* heart rate, *LGE* late gadolinium enhancement, *LV* left ventricle, *MI* myocardial infarction, *PCI* percutaneous coronary intervention, *RPP* rate-pressure product (pressure mmHg x Heart rate bpm)/1000, *RV* right ventricle, *SD* standard deviation

^a^BMI ≥ 30 kg/m^2^

^b^Glomerular filtration rate < 60 ml/min/1.73 m^2^

^c^To detect myocardial ischemia in asymptomatic patient every 3 to 5 years in accordance with ESC guidelines. (REF)

^d^Coronary stenosis of unknown significance on CCTA

^e^Presence of LGE with < 50% transmurality

Patients with myocardial ischemia were older (69.0 ± 9.3 vs. 67.7 ± 10.7 years, p = 0.003) and more frequently male (85.1% vs. 80.8%, p = 0.024). Of the 376 patients with myocardial ischemia, 267 (71.0%) had a coronary angiography with early revascularization < 90 days after CMR. Among those, 11 patients were censored due to the recurrence of MI or cardiovascular mortality within 90 days after CMR.

### CMR study

Of 1529 asymptomatic patients with prior obstructive CAD, 1,466 (95.9%) completed the stress CMR protocol. Reasons for failure to complete CMR were claustrophobia (1.4%), intolerance to stress agent (0.7%), renal failure (0.7%), poor gating (0.7%) and declining participation (0.6%) (Additional file 1). No patient died during or shortly after CMR in relation with the study. There were two cases of unstable angina, one case of acute pulmonary edema and one patient with persistent atrial fibrillation. Detailed safety results are presented in Additional file 1.

### Prognostic value

**Fig. 2:**
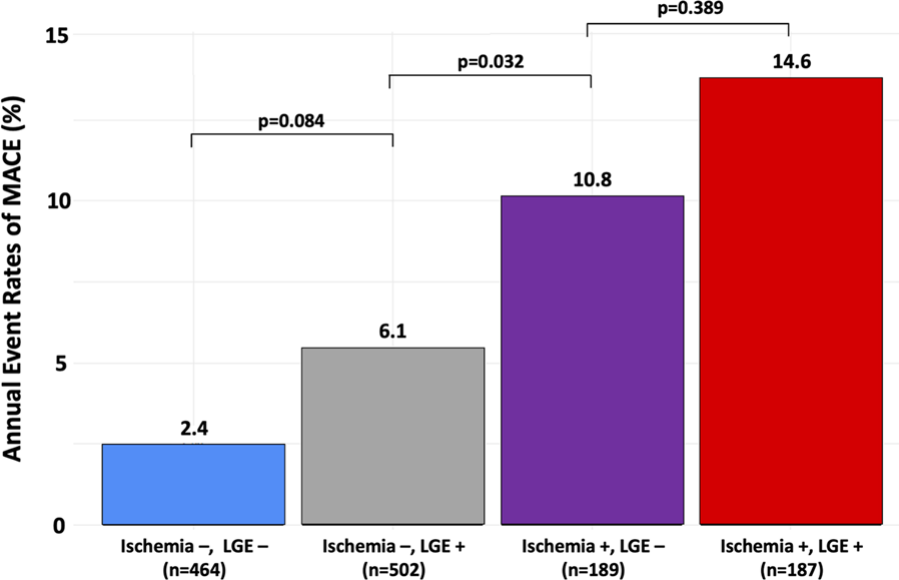
Annualized rates of MACE stratified by the presence of myocardial ischemia and LGE. Annual event rates (N = 1342) of MACE (cardiovascular mortality and non-fatal MI) for the entire study cohort

**Fig. 3:**
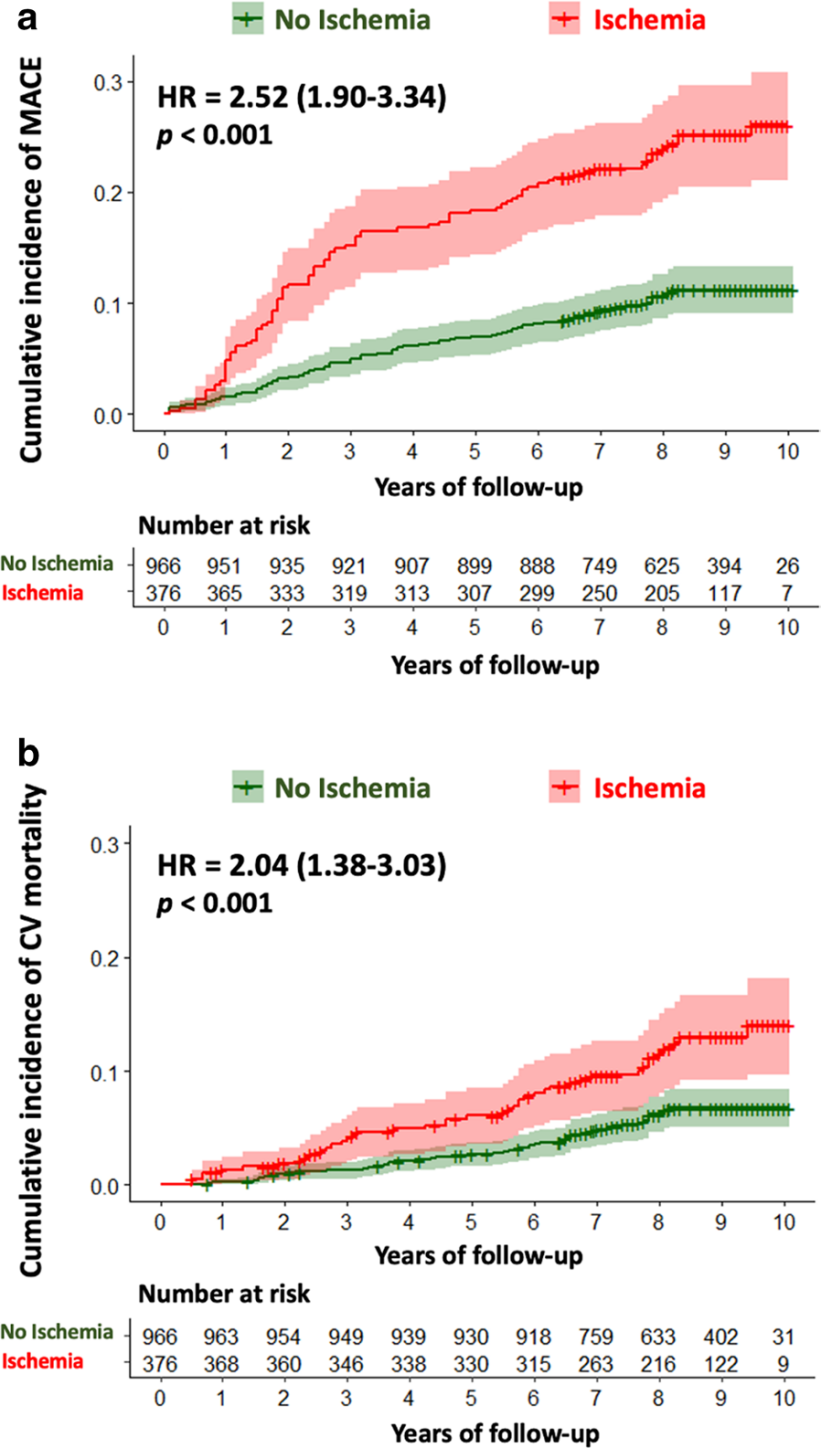
Kaplan–Meier curves for MACE and cardiovascular mortality. Kaplan Meier curves of MACE (cardiovascular mortality or non-fatal MI) (**a**) and cardiovascular mortality (**b**) as a function of length of follow-up in patients with and without myocardial ischemia

**Fig. 4:**
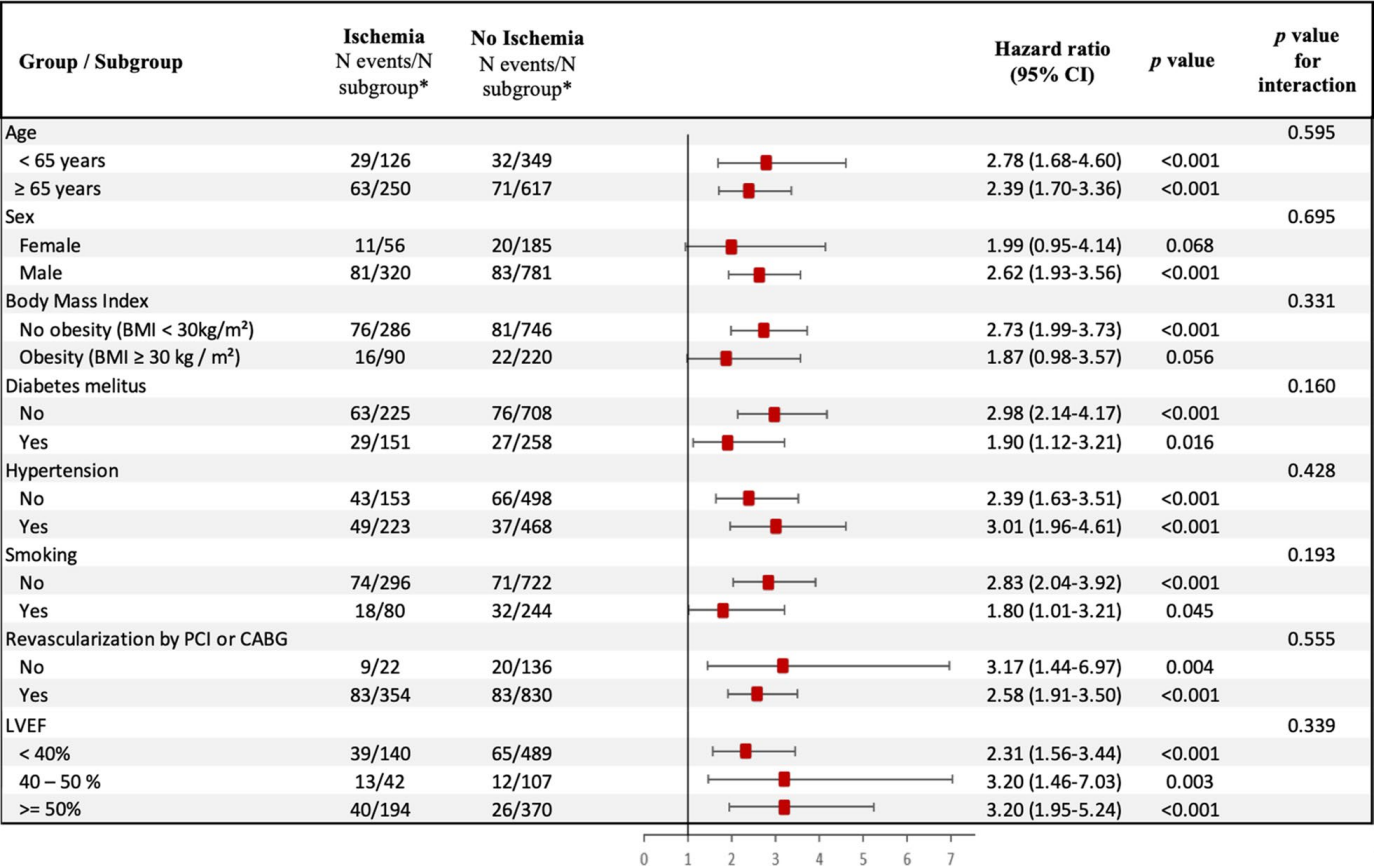
Subgroup analysis. Forest-plot of incidence of MACE based on the presence of myocardial ischemia in prespecified subgroups. *N events/N subgroup: number of patients had a major adverse clinical events (MACE)/number of patients in the subgroup

Median (IQR) follow-up was 8.3 (7.0–9.4) years. There were 195 MACE (13.3%), including 102 cardiovascular mortality (7.6%) and 93 non-fatal MI (6.9%). Furthermore, 190 all-cause mortality (14.2%), 172 late coronary revascularizations without emergency (12.8%) (6 CABG), 78 hospitalizations for heart failure (5.8%), and 23 sustained documented ventricular tachycardia (1.6%) were recorded. Annualized event rates were 6.7% for MACE, 2.3% for cardiovascular mortality, and 6.6% for all-cause mortality. The annualized event rates for MACE according to the presence or absence of myocardial ischemia and LGE are presented in Fig. 2. Patients without myocardial ischemia or LGE had a lower annualized rate of MACE (2.4%), whereas the annualized rate of MACE was greater for patients with both myocardial ischemia and LGE (14.6%) (p < 0.001). The annualized rates of MACE and cardiovascular mortality are depicted in Additional file 1 based on the presence and extent of myocardial ischemia. The annualized rate of MACE was higher for patients with moderate or severe ischemia than patients with mild ischemia (16.8%, 42.2% and 7.3%, respectively; p_trend_ < 0.001). The univariable analysis of baseline individuals and CMR characteristics for the prediction of MACE and cardiovascular mortality is shown in Table 2. Age, hypertension, the presence of myocardial ischemia, the number of ischemic segments, the presence of LGE, LVEF and both LV end-diastolic and end-systolic volumes indexed were all significantly associated with MACE. Using Kaplan–Meier analysis, myocardial ischemia and LGE were associated with the occurrence of MACE (HR 2.52; 95% CI 1.90–3.34 and HR 2.04; 95% CI 1.38–3.03, respectively; both p < 0.001) (Fig. 3). In addition, myocardial ischemia was associated with cardiovascular mortality (HR 2.52; 95% CI 1.90–3.34), non-fatal MI (HR 3.09; 95% CI 2.06–4.64), late coronary revascularization (HR 2.30; 95% CI 1.45–3.66) (all p < 0.001) and all-cause mortality (HR 1.55; 95% CI 1.15–2.08, p = 0.004; Additional file 1). The prognostic value of myocardial ischemia or LGE to predict MACE was not significantly different in men and women (p = 0.695, Fig. 4).

**Table 2:** Univariable analysis of clinical and CMR characteristics for prediction of adverse events (N = 1342)

	MACE		Cardiovascular Mortality	
	Hazard ratio (95% CI)	p value	Hazard ratio (95% CI)	p value
Age	1.02 (1.01–1.03)	0.007	1.05 (1.03–1.07)	< 0.001
Male	1.18 (0.80–1.73)	0.400	1.28 (0.74–2.22)	0.374
Body mass index	0.96 (0.93–1.00)	0.123	0.98 (0.93–1.01)	0.382
Hypertension	0.73 (0.55–0.97)	0.031	0.74 (0.50–1.09)	0.127
Diabetes mellitus	0.91 (0.67–1.25)	0.575	1.14 (0.75–1.71)	0.546
Dyslipidemia	0.89 (0.66–1.18)	0.408	0.76 (0.51–1.12)	0.168
Current or previous smoking	1.09 (0.79–1.51)	0.594	0.96 (0.61–1.52)	0.861
Family history of CAD	0.88 (0.64–1.19)	0.401	1.04 (0.69–1.57)	0.846
Prior PCI	0.85 (0.64–1.13)	0.263	0.56 (0.38–0.83)	0.003
Prior CABG	0.88 (0.64–1.19)	0.401	1.04 (0.69–1.57)	0.846
Stroke	0.76 (0.28–2.05)	0.592	0.74 (0.18–2.99)	0.669
Renal Failure	0.42 (0.06–3.00)	0.387	0.00 (0.00– + ∞)	0.993
Peripheral atheroma	0.81 (0.47–1.39)	0.442	1.01 (0.51–2.00)	0.975
Heart failure hospitalization	1.40 (0.62–3.15)	0.422	1.84 (0.68–5.01)	0.231
Presence of myocardial ischemia	2.52 (1.90–3.34)	< 0.001	2.04 (1.38–3.03)	< 0.001
Number of segments of myocardial ischemia	1.47 (1.39–1.56)	< 0.001	1.40 (1.29–1.53)	< 0.001
Presence of LGE	1.66 (1.13–2.42)	0.009	1.87 (1.08–3.24)	0.025
Number of segments of LGE	1.35 (1.26–1.45)	< 0.001	1.38 (1.25–1.53)	< 0.001
LVEF, per 10%	0.84 (0.75–0.94)	0.003	0.88 (0.75–1.03)	0.101
LV end-diastolic volume index, per 10 ml/m^2^	1.06 (1.02–1.11)	0.006	1.05 (0.98–1.11)	0.150
LV end-systolic volume index, per 10 ml/m^2^	1.09 (1.03–1.14)	0.001	1.07 (1.00–1.15)	0.052
RV ejection fraction, %	0.92 (0.75–1.15)	0.490	1.05 (0.78–1.48)	0.980

*CABG* coronary artery bypass grafting, *CAD* coronary artery disease, *CI* confidence interval, *CMR* cardiovascular magnetic resonance, *HF* heart failure, *LGE* late gadolinium enhancement, *LV* left ventricle, *LVEF* left ventricular ejection fraction, *MACE* major adverse cardiac events, *MI* myocardial infarction, *PCI* percutaneous intervention, *RV* right ventricle

**Fig. 5:**
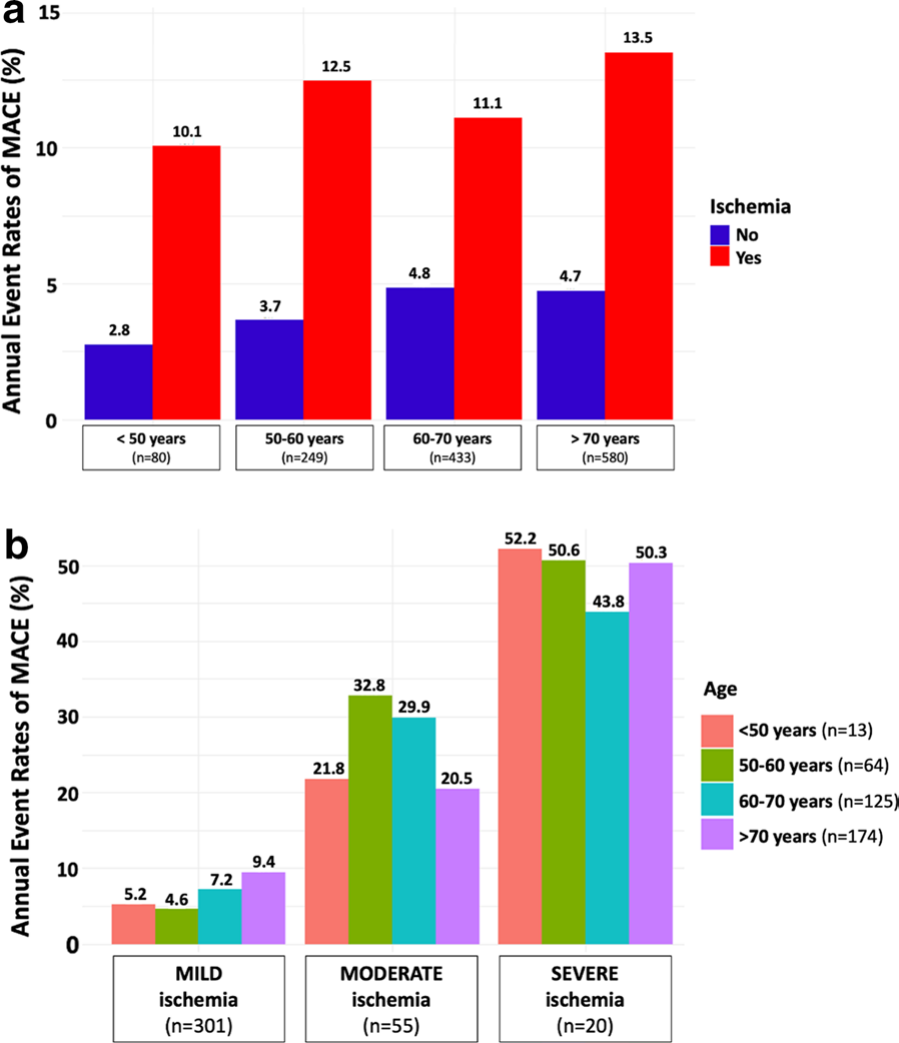
Annualized event rates of MACE stratified by age, presence/absence and the amount of myocardial ischemia. Annualized event rates of MACE are stratified by presence/absence of myocardial ischemia (**a**) and the amount of myocardial ischemia (**b**) in different age categories: < 50 years; 50–60 years; 60–70 years and > 70 years. Mild, moderate, and severe myocardial ischemia were defined as the involvement of 1–2, 3–5, and ≥ 6 myocardial segments, respectively

In multivariable stepwise Cox regression (model 2), the presence of myocardial ischemia and LGE were independent predictors of a higher incidence of MACE (HR 2.80 95% CI 2.10–3.73, *p* < 0.001 and HR 1.51; 95% CI 1.01–2.27, *p* = 0.045; respectively) and cardiovascular mortality (HR 2.20 95% CI 1.47–3.28, *p* < 0.001 and HR 2.07; 95% CI 1.13–3.78, *p* = 0.018; respectively) (Table 3). In competitive risk analysis, the presence of myocardial ischemia was independently associated with non-fatal MI and cardiovascular mortality (both p < 0.001) (Additional file 1). The prognostic value of myocardial ischemia remained consistent in different subsamples of clinical interest such as diabetics and non-diabetics (**Fig. ****4**). The presence and extent of myocardial ischemia remained associated with MACE in each age category (**Fig. ****5**).

**Table 3:** Multivariable cox regression analysis for the prediction of adverse events (N = 1342)

	MACE		Cardiovascular Mortality	
	Hazard ratio (95% CI)	p value	Hazard ratio (95% CI)	p value
Model 1^a^				
Age	1.03 (1.01–1.04)	< 0.001	1.06 (1.04–1.08)	< 0.001
Hypertension	0.72 (0.54–0.96)	0.026	0.70 (0.47–1.04)	0.073
Dyslipidemia	–	–	0.76 (0.51–1.12)	0.168
Prior PCI	–	–	0.51 (0.34–0.76)	0.001
LVEF, per 10%	0.87 (0.78–0.97)	0.028	0.89 (0.76–1.05)	0.211
LV end-systolic volume index, per 10 ml/m^2^	1.10 (1.05–1.16)	< 0.001	1.13 (1.05–1.21)	< 0.001
Model 2^b^				
Presence of myocardial ischemia	2.80 (2.10–3.73)	< 0.001	2.20 (1.47–3.28)	< 0.001
Presence of LGE	1.51 (1.01–2.27)	0.045	2.07 (1.13–3.78)	0.018
Model 3^c^				
Age	1.03 (1.01–1.04)	< 0.001	1.06 (1.03–1.08)	< 0.001
Male	1.17 (0.79–1.72)	0.424	1.25 (0.72–2.17)	0.424
Body mass index	0.98 (0.94–1.00)	0.233	0.95 (0.63–1.52)	0.851
Hypertension	0.72 (0.53–0.98)	0.037	0.68 (0.44–1.03)	0.068
Diabetes mellitus	0.95 (0.69–1.31)	0.748	1.23 (0.80–1.88)	0.344
Dyslipidemia	0.97 (0.72–1.31)	0.831	0.78 (0.52–1.19)	0.251
Current or previous smoking	1.13 (0.81–1.58)	0.477	1.17 (0.72–1.88)	0.523
LVEF, per 10%	0.81 (0.72–0.92)	< 0.001	0.83 (0.70–0.98)	0.024
Model 4^d^				
Presence of myocardial ischemia	2.85 (2.13–3.81)	< 0.001	2.16 (1.44–3.24)	< 0.001
Presence of LGE	0.98 (0.97–0.99)	0.005	1.89 (1.05–3.41)	0.034

^a^Covariates in the model 1 by stepwise variable selection with entry and exit criteria set at the p ≤ 0.2 level:

for MACE: age, hypertension, LVEF per 10% and LV end-systolic volume index, per 10 ml/m^2^.

for CV mortality: age, hypertension, dyslipidemia, previous PCI, LVEF per 10% and LV end-systolic volume index, per 10 ml/m^2^.

^b^Covariates in the model 2: model 1 + presence of myocardial ischemia and LGE

^c^Covariates in the model 3 were traditional cardiovascular risk factors: age, male, BMI, hypertension, diabetes mellitus, current or previous smoking, dyslipidemia and LVEF per 10%

^d^Covariates in the model 4: model 3 + presence of myocardial ischemia and LGE

*CI* confidence interval, *EDVi* end-diastolic volume index, *ESVi* end-systolic volume index, *HR* hazard ratio, *LGE* late gadolinium enhancement, *MACE* major adverse cardiac events, *LV* left ventricle, *LVEF* left ventricular ejection fraction

### Incremental prognostic value of CMR

Model 1 with stepwise variable selection had a baseline C statistic value of 0.61 (95% CI 0.57–0.64) for predicting MACE, whereas model 3 including traditional cardiovascular risk factors had a C statistic value of 0.68 (95% CI 0.61–0.71). The addition of CMR–induced myocardial ischemia and LGE significantly improved the C statistic of model 1 to 0.68 (95% CI 0.62–0.71; C statistic improvement for model 1: 0.07; NRI = 0.207; IDI = 0.021) and of model 3 to 0.72 (95% CI 0.67–0.78; C statistic improvement for model 2: 0.04; NRI = 0.359; IDI = 0.065) (Additional file 1).

## Discussion

In asymptomatic patients with known CAD referred for stress CMR, the presence of myocardial ischemia and LGE were independent long-term predictors of MACE and cardiovascular mortality. Furthermore, the presence of myocardial ischemia and LGE improved model discrimination in predicting MACE, after adjusting for covariates or traditional cardiovascular risk factors.

The prevalence of myocardial ischemia and LGE was 28.0% and 51.3%, respectively, which is consistent with previous studies in patients with known CAD [4, 13]. In agreement with others, the prevalence of myocardial ischemia was higher in diabetics (36.9%) [24, 25]. Of note, myocardial ischemia and LGE remained good prognosticators in different clinical subgroups, including diabetic and non-diabetic patients, obese and nonobese patients, with or without prior revascularization, and regardless of sex or LVEF, thereby extending the aggregate data on the prognostic value of stress CMR [9, 10, 17, 18]. Besides the presence of myocardial ischemia, the current report shows the prognostic value of the extent of myocardial ischemia in these patients, which concur with previous reports [13]. Similar to other studies, the presence of LGE was an independent predictor of MACE in the current study [26]. Moreover, the safety profile of stress CMR was excellent, as described in a registry of > 11,000 patients with a significant proportion of asymptomatic patients with prior CAD [27].

Whereas the interest of coronary revascularization has been recently debated in patients with stable CAD [3], some studies have suggested potential clinical interest of coronary revascularization in asymptomatic patients with documented myocardial ischemia [28]. A large study of 1,473 patients with objective myocardial ischemia showed a significant reduction in cardiovascular mortality at > 5 years in the revascularization group as compared with the medical therapy group (25 vs. 34%) [29]. Interestingly, patients who received initial revascularization for myocardial ischemia and who had documented residual ischemia on follow-up stress testing had a higher cardiovascular mortality rate [30]. Although the current guidelines do not recommend systematic stress testing in asymptomatic patients with prior CAD, the current data show the incremental prognostic value of stress perfusion CMR over traditional risk factors in these patients. Recent studies have shown promizing new therapy strategies targeting inflammation and coagulation to decrease the risk of recurrent CV events in patients with CAD [30, 31]. An improved risk stratification using stress CMR could allow to identify high-risk patients who could benefit from treatment intensification, new therapy and/or revascularization.

### Study limitations

Our study has several limitations. First, 124 (8.5%) patients were lost to follow-up, which can be explained by a relatively long follow-up and the design of the study. However, the French National Registry of Death was carefully reviewed, which strengthens the mortality data. The analysis of the CMR perfusion scans was visual but it represents the most widely accepted clinical method with optimal diagnostic accuracy. Although adenosine is commonly used for stress perfusion CMR, dipyridamole was used in our center between 2009 and 2011 mainly because of medico-economic reasons and similar or very close efficacy/safety profile compared to adenosine.

## Conclusions

In this large monocenter study, vasodilator stress perfusion CMR has accurate discriminative long-term prognostic value in asymptomatic patients with known CAD. Myocardial ischemia and LGE are independently associated with cardiovascular mortality or non-fatal MI over a long-term follow-up and offer incremental prognostic value over traditional CAD risk factors. The clinical implications of improved risk stratification on diagnostic and therapeutic decision making remain to be evaluated in this population.

## Supplementary Information

**Additional file 1: Supplement 1:** Study flowchart. **Supplement 2:** Safety results. **Supplement 3:** Annualized event rates of MACE (A) and Cardiovascular mortality (B) stratified by the extent of myocardial ischemia (N = 1342). **Supplement 4:** Table. Univariable analysis of myocardial ischemia for prediction of adverse events (N = 1342). **Supplement 5:** Competitive risk analysis. **Supplement 6:** Table. Univariable and Multivariable Competing Risk Regression Analysis (N = 1342). **Supplement 7:** Table. Discrimination and reclassification associated with myocardial ischemia and LGE for prediction of MACE (N = 1342).

## Data Availability

All data generated or analysed during this study are included in this published article [and its supplementary information files].

## Abbreviations

BMI: Body mass index
bSSFP: Balanced steady state free precession
CABG: Coronary artery bypass grafting
CAD: Coronary artery disease
CI: Confidence interval
CIF: Cumulative incidence function
CMR: Cardiovascular magnetic resonance
ECG: Electrocardiogram
HR: Hazard ratio
IDI: Integrative discrimination index
LGE: Late gadolinium enhancement
LV: Left ventricle/left ventricular
LVEF: Left ventricular ejection fraction
MACE: Major adverse cardiovascular event
MI: Myocardial infarction
NRI: Net reclassification index
PCI: Percutaneous coronary intervention.
RPP: Rate pressure product
SD: Standard deviation
