# Supplementary material for: Prognostic value of stress cardiovascular magnetic resonance in asymptomatic patients with known coronary artery disease
Source: J Cardiovasc Magn Reson. 2021 Mar 8;23:19. doi: 10.1186/s12968-021-00721-8 (PMC7938489; doi:10.1186/s12968-021-00721-8)
Supplement: Supplementary file 1 — Additional file 1: Supplement 1: Study flowchart. Supplement 2: Safety results. Supplement 3: Annualized event rates of MACE (A) and Cardiovascular mortality (B) stratified by the extent of myocardial ischemia (N = 1342). Supplement 4: Table. Univariable analysis of myocardial ischemia for prediction of adverse events (N = 1342). Supplement 5: Competitive risk analysis. Supplement 6: Table. Univariable and Multivariable Competing Risk Regression Analysis (N = 1342). Supplement 7: Table. Discrimination and reclassification associated with myocardial ischemia and LGE for prediction of MACE (N = 1342). [file 12968_2021_721_MOESM1_ESM.docx]

**SUPPLEMENTAL MATERIEL**

**Table of Contents:**

- Supplement 1: Figure. Study flowchart.
- Supplement 2: Safety results.
- Supplement 3: Figure. Annualized event rates of MACE (A) and Cardiovascular mortality (B) stratified by the extent of myocardial ischemia (N=1,342).
- Supplement 4: Table. Univariable analysis of myocardial ischemia for prediction of adverse events (N=1,342).
- Supplement 5: Figure. Competitive risk analysis.
- Supplement 6: Table. Univariable and Multivariable Competing Risk Regression Analysis (N=1,342).
- Supplement 7: Table. Discrimination and reclassification associated with myocardial ischemia and LGE for prediction of MACE (N=1,342).

**SUPPLEMENT 1**

**Figure. Study flowchart.**

**
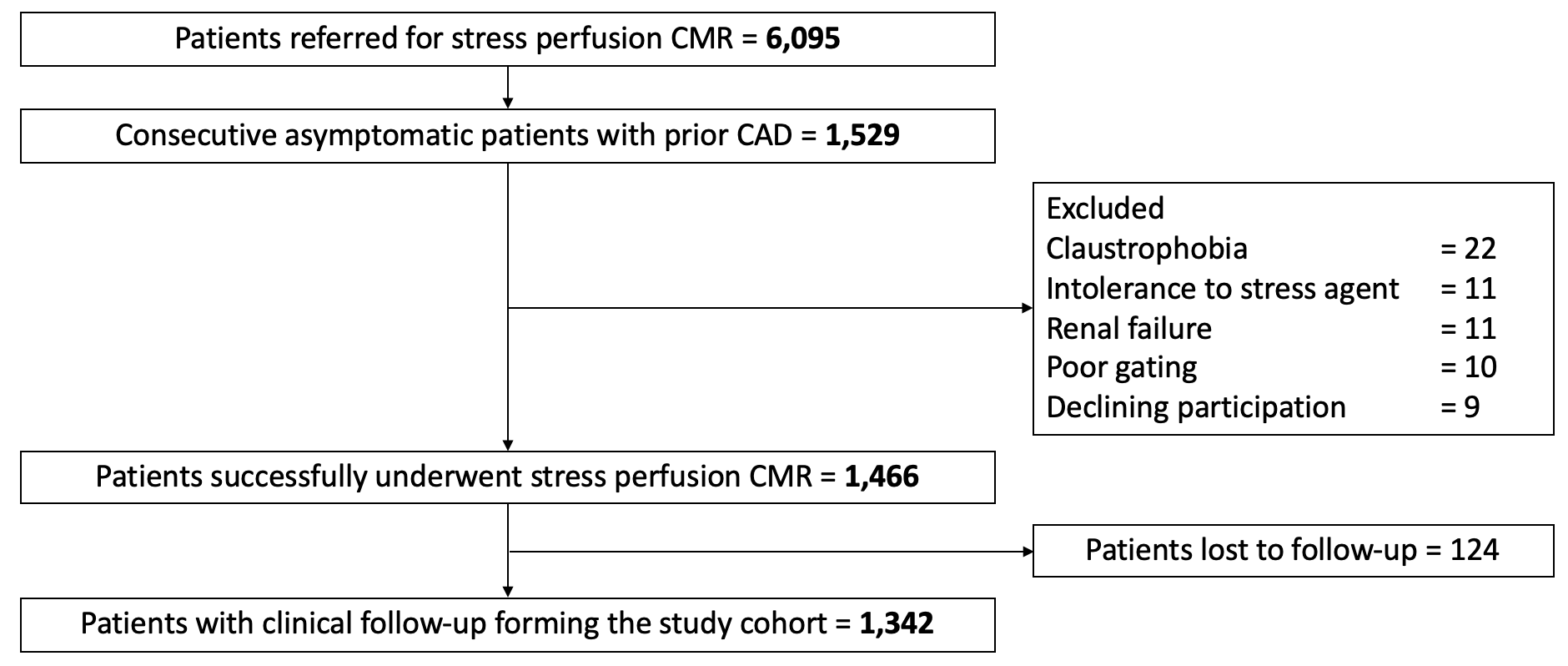
**

**SUPPLEMENT 2**

**Safety results**

There were two cases of unstable angina, one acute pulmonary edema and one patient with persistent atrial fibrillation, but no transient ischemic attack, disabling stroke, ST elevation MI or sustained ventricular tachycardia were recorded in relation to stress CMR. The main adverse events during or immediately after the study were as follows: 254 headaches (17.3%), 177 nausea or vomiting (12.1%), 164 chest discomfort due to dipyridamole (11.2%), 33 angina with ECG evidence of myocardial ischemia (2.3%), and 32 dizziness (2.2%). For all patients, symptoms resolved quickly with intravenous theophylline and additional sublingual nitrates and/or intravenous betablockers in 11 patients (0.8%).

**SUPPLEMENT 3**

**Figure. Annualized rates of MACE (A) and Cardiovascular mortality (B) stratified by the extent of myocardial ischemia (N=1,342).**

**
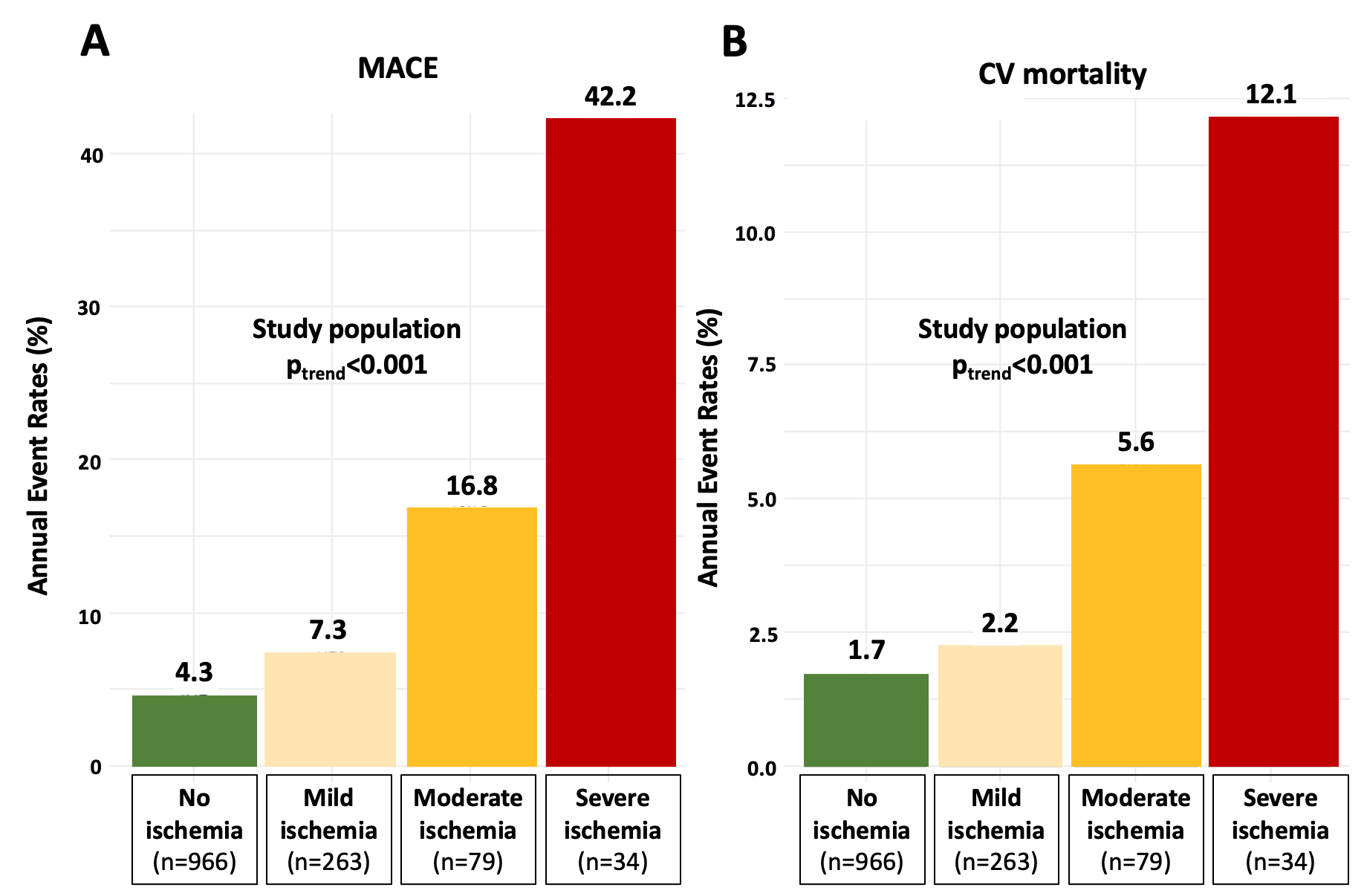
**Mild, moderate, and severe ischemia were defined as the involvement of 1–2, 3–5, and ≥ 6 myocardial segments, respectively (14). Test comparing the groups was based on the Cochran-Armitage test for trend.

**Supplement 4**

**Table. Univariable analysis of CMR-induced myocardial ischemia for prediction of adverse events (N=1,342).**

|  | **Univariable analysis** | |  |
| --- | --- | --- | --- |
|  | **Hazard Ratio**  **(95% CI)** | **p value** |  |
|  |  |  |  |
| ***Primary outcome (MACE)*** |  |  |  |
| Cardiovascular mortality | 2.04 (1.38-3.03) | **<0.001** |  |
| Non-fatal MI | 3.09 (2.06-4.64) | **<0.001** |  |
|  |  |  |  |
| ***Secondary outcomes*** |  |  |  |
| All-cause of mortality | 1.55 (1.15-2.08) | **0.004** |  |
| Late coronary revascularization without emergency | 2.30 (1.45-3.66) | **<0.001** |  |
| Hospitalization for heart failure | 1.43 (0.89-2.28) | 0.140 |  |
| Ventricular tachycardia | 1.80 (0.78-4.15) | 0.171 |  |
|  |  |  |  |

*Abbreviations*: CI: confidence interval; HR: hazard ratio; LGE: late gadolinium enhancement; MACE: major adverse cardiac events; MI: myocardial infarction.

**SUPPLEMENT 5**

**Figure. Competitive risk analysis.**

**
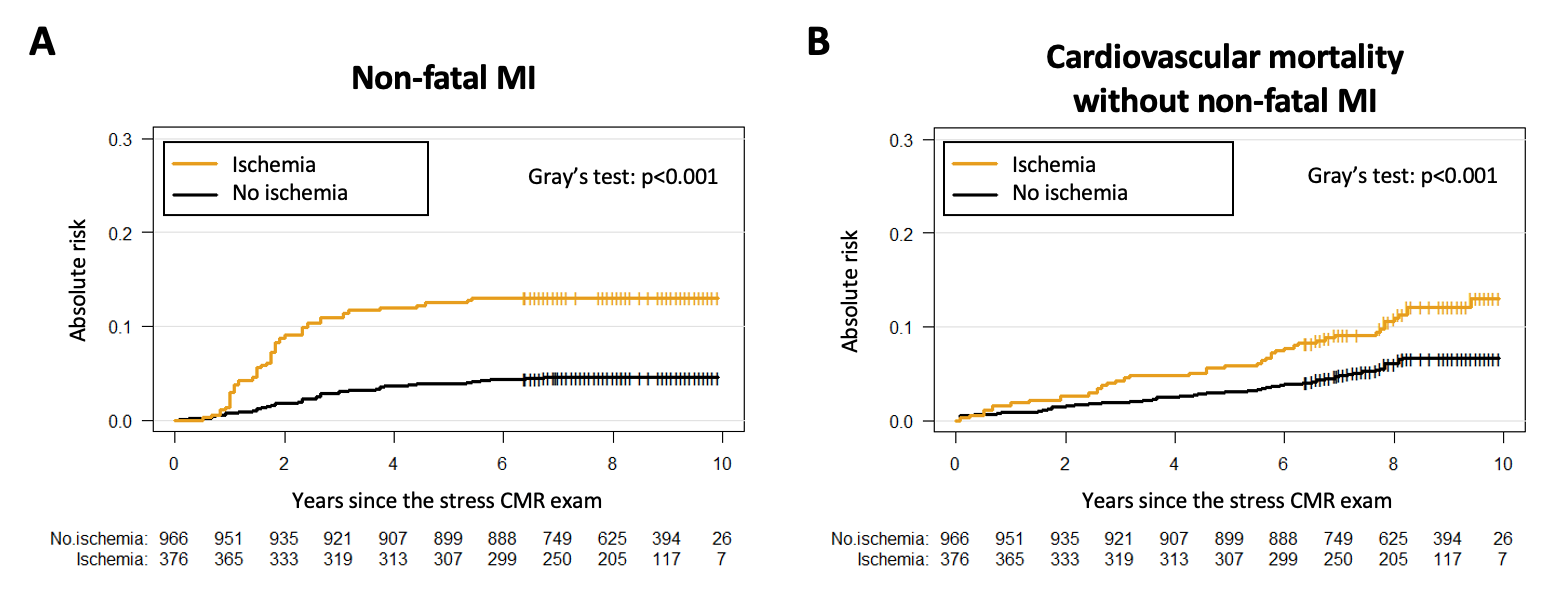
**Cumulative incidence functions of non-fatal MI (A) or cardiovascular mortality without non-fatal MI (B).

**Supplement 6**

**Table. Univariable and Multivariable Competing Risk Regression Analysis (N=1,342).**

| **Non-fatal MI Cardiovascular Mortality** | | | | | | | | | | |  |
| --- | --- | --- | --- | --- | --- | --- | --- | --- | --- | --- | --- |
|  |  |  |  |  |  |  |  |  |  |  |  |
|  | **Univariable analysis** | |  | **Multivariable analysis** | |  | **Univariable analysis** | |  | **Multivariable analysis** | |
|  | **sHR* (95% CI)** | **p value** |  | **sHR* (95% CI)** | **p value** |  | **sHR* (95% CI)** | **p value** |  | **sHR* (95% CI)** | **p value** |
|  |  |  |  |  |  |  |  |  |  |  |  |
| Age | 0.99 (0.98-1.01) | 0.46 |  | - | - |  | 1.05 (1.02-1.07) | **<0.001** |  | 1.05 (1.03-1.08) | **<0.001** |
| Male | 1.06 (0.62-1.81) | 0.83 |  | - | - |  | 1.29 (0.74-2.23) | 0.37 |  | - | - |
| Body mass index | 1.02 (0.97-1.07) | 0.51 |  | - | - |  | 0.96 (0.91-1.00) | 0.067 |  | - | - |
| Diabetes melitus | 0.70 (0.43-1.12) | **0.14** |  | 0.60 (0.36-1.01) | 0.054 |  | 1.16 (0.76-1.74) | 0.49 |  | - | - |
| Hypertension | 0.74 (0.49-1.11) | **0.15** |  | 0.72 (0.47-1.11) | 0.140 |  | 0.74 (0.50-1.10) | 0.13 |  | 0.62 (0.42-0.92) | **0.019** |
| Dyslipidemia | 1.06 (0.69-1.61) | 0.8 |  | - | - |  | 0.76 (0.52-1.13) | 0.17 |  | - | - |
| Smoking | 1.23 (0.78-1.94) | 0.36 |  | - | - |  | 0.96 (0.60-1.51) | 0.85 |  | - | - |
| Prior MI | 0.88 (0.59-1.33) | 0.55 |  | - | - |  | 1.70 (1.12-2.57) | **0.012** |  | 1.62 (1.00-2.61) | **0.047** |
| History of hospitalization for HF | 0.91 (0.23-3.57) | 0.89 |  | - | - |  | 1.86 (0.68-5.09) | 0.23 |  | - | - |
| Presence of myocardial ischemia | 3.02 (2.02-4.54) | **<0.001** |  | 3.48 (0.29-5.28) | **<0.001** |  | 1.93 (1.30-2.86) | **<0.001** |  | 2.03 (1.38-2.99) | **<0.001** |
| Presence of LGE | 1.44 (0.85-2.43) | **0.18** |  | 1.60 (0.95-2.69) | 0.079 |  | 1.86 (1.07-3.21) | **0.027** |  | 1.63 (0.88-3.02) | 0.12 |
| LVEF | 0.98 (0.96-0.99) | **0.012** |  | - | - |  | 0.99 (0.97-1.00) | 0.12 |  | - | - |
|  |  |  |  |  |  |  |  |  |  |  |  |

* HR of the subdistribution hazard function

^†^ Covariates in the model: traditional cardiovascular risk factors: age, male, BMI, hypertension, diabetes mellitus, current or previous smoking, dyslipidemia and LVEF per 10%.

*Abbreviations: CI: confidence interval; LGE: late gadolinium enhancement; LV: left ventricle; LVEF: left ventricular ejection fraction.*

**Supplement 7**

**Table. Discrimination and reclassification associated with myocardial ischemia and LGE for prediction of MACE (N=1,342).**

|  | **MACE** | | | |
| --- | --- | --- | --- | --- |
|  | **C-index**  **(95%CI)** | **NRI**  **(95%CI)** | **IDI**  **(95%CI)** |  |
|  |  |  |  |  |
| Model 1 (stepwise selection) * | 0.61 (0.57-0.64) | Reference | Reference |  |
| Model 2 (model 1 + myocardial ischemia and LGE) ^†^ | 0.68 (0.62-0.71) | 0.207 (0.108-0.306) | 0.021 (0.007-0.035) |  |
|  |  |  |  |  |
| Model 3 (traditional risk factors) ^‡^ | 0.68 (0.61-0.71) | Reference | Reference |  |
| Model 4 (model 3 + myocardial ischemia and LGE) ^§^ | 0.72 (0.67-0.78) | 0.359 (0.257-0.461) | 0.065 (0.047-0.083) |  |
|  |  |  |  |  |

* Covariates in the **model 1** by stepwise variable selection with entry and exit criteria set at the p≤0.2 level:

- for MACE: age, hypertension, LVEF per 10% and LV end-systolic volume index, per 10 ml/m^2^.
- for cardiovascular mortality: age, hypertension, dyslipidemia, previous PCI, LVEF per 10% and LV end-systolic volume index, per 10 ml/m^2^.

^†^ Covariates in the **model 2**: model 1 + presence of myocardial ischemia and LGE.

^‡^ Covariates in the **model 3** were traditional cardiovascular risk factors: age, male, BMI, hypertension, diabetes mellitus, current or previous smoking, dyslipidemia and LVEF per 10%.

^§^ Covariates in the **model 4**: model 3 + presence of myocardial ischemia and LGE.

*Abbreviations: CI: confidence interval; IDI:* integrative discrimination index; *LGE: late gadolinium enhancement; MACE: major adverse cardiac events; NRI:* net reclassification improvement.
